# Impact of altered environment and early postnatal methamphetamine exposure on serotonin levels in the rat hippocampus during adolescence

**DOI:** 10.1186/s42826-024-00192-9

**Published:** 2024-02-02

**Authors:** Barbora Čechová, Jana Jurčovičová, Ivana Petríková, Šimon Vaculín, Štěpán Šandera, Romana Šlamberová

**Affiliations:** Department of Physiology, Third Faculty of Medicine, Charles University, Prague, Czech Republic

**Keywords:** Methamphetamine, Serotonin, Adolescence, Enriched environment, Hippocampus

## Abstract

**Background:**

Methamphetamine (MA) is a highly abused psychostimulant across all age groups including pregnant women. Because developing brain is vulnerable by the action of drugs, or other noxious stimuli, the aim of our study was to examine the effect of early postnatal administration of MA alone or in combination with enriched environment (EE) and/or stress of separate housing, on the levels of serotonin (5HT) in the hippocampus of male rat pups at three stages of adolescence (postnatal day (PND) 28, 35 and 45). MA (5 mg/kg/ml) was administered subcutaneously (sc) to pups (direct administration), or via mothers' milk between PND1 and PND12 (indirect administration). Controls were exposed saline (SA). Pups were exposed to EE and/or to separation from the weaning till the end of the experiment.

**Results:**

On PND 28, in sc-treated series, EE significantly increased the muted 5HT in SA pups after separation and restored the pronounced inhibition of 5HT by MA. No beneficial effect of EE was present in pups exposed to combination of MA and separation. 5HT development declined over time; EE, MA and separation had different effects on 5HT relative to adolescence stage.

**Conclusions:**

Present study shows that MA along with environment or housing affect 5HT levels, depending on both the age and the method of application (direct or indirect). These findings extend the knowledge on the effects of MA alone and in combination with different housing conditions on the developing brain and highlight the increased sensitivity to MA during the first few months after birth.

## Background

Methamphetamine (MA) is one of the most abused synthetic psychostimulants. Globally, it is the second most popular illicit drug of abuse after cannabis. The fact that it quickly produces several desired effects, including alertness, increased energy, and decreased appetite, makes it very popular also in addicted pregnant women [1, 2]. There are several consequences associated with chronic exposure to this drug. Among the most resonating effects are neurological and psychiatric complications. A significant dysfunction of neurotransmitter (NT) systems in the brain causes a negative impact on the central nervous system (CNS). Markers of MA-induced neurotoxicity include, among others, degeneration of 5HT nerve endings, long-term depletions of 5HT content as well as decreases in the transporters for 5HT within the brain. Proper 5HT neurotransmission is essential for behavioral and emotional balance, such as stable moods, regular sleep, and anxiety control [3]. This NT also performs a crucial role in the brain’s organization, and its disruption can lead to social interaction deficits and psychiatric problems [4]. Abnormalities in this system during critical periods of development have various consequences, such as altered anxiety responses and deepened depression. The number of serotonergic synapses is constantly changing during development. 5HT synapses in the brain reach adult levels in the early postnatal days, and then, in the rat forebrain, these levels can decrease during adolescence [5, 6]. 5HT turnover is reportedly several times lower during mid-adolescence than in pre-adolescent or post-adolescent rats [6]. Immature 5HT tissue content and lower 5HT release in adolescents may also be caused by lower 5HT stores in the terminals of serotonergic neurons [7, 8]. 5HT depletion during adolescence in female mice has been suggested to be protective against heightened anxiety and excessive use of drugs such as alcohol or other recreational drugs [3].

Several abnormalities within 5HT system have been reported in the hippocampus (HP). In humans, this brain region linearly matures between week 21 and 31 as reported by study using in vivo magnetic resonance examination [9]. During its development in utero, HP is vulnerable to stress, undernutrition, and metabolic disturbance, which may result in reduction of the HP volume that is associated with impaired learning and memory in children [9, 10]. During above mentioned period there is the increase in levels of 5HT which promotes dendritic maturation [11]. In rodents, this brain region matures during first two postnatal weeks [6], which corresponds to the third trimester of a human pregnancy [12, 13].

Since MA is lipophilic, it crosses the placenta and passes into breast milk, that both may affect development of the offspring in a very alarming way [14]. Prenatal MA exposure disrupts limbic integrity and changes the microstructure of white matter compared to unexposed children [15]. Our laboratory previously showed impairments due to prenatal MA exposure persisting until adulthood [16]. Prenatal exposure to MA altered pain sensitivity [17] and seizure response [18] and decreased social interactions in adult rats [13, 14, 19]. Our lab also showed that early MA exposure affected cognitive functions such as learning and memory and postnatal MA exposure impaired the performance of females during water maze testing [13]. Since both prenatal and neonatal MA exposure causes severe behavioral impairments, there have been reports that proper maternal care can reverse those effect and pups raised by control mother during lactation had better results in behavioral testing [14]. Since study by Petríková-Hrebíčková (2021) yielded the finding that early postnatal MA exposure (PND 1–11) impairs performance of adult rats in Morris Water Maze task in terms of increased searched errors, we decided to explore this developmental period further. Our laboratory also previously reported that pups raised by mother exposed to MA during lactation (pups were exposed indirectly by breastfeeding) stunted in development in comparison to pups raised by control mothers [20]. So far, we have mainly investigated the long-term changes caused by developmental exposure to MA persisting into adulthood, but the impact on adolescence, one of the most critical periods in an individual's development, has not been sufficiently explored yet. Major developmental processes occur during adolescence, which is typically the period between 12 and 20 years of age in humans, and postnatal days (PNDs) are 28–45 in rodents, however the boundaries of this period are still unclear [21].

As mentioned above, role of proper maternal care on pup development leads to the question: how important is the environment during this period? While a stressful situation can have a negative effect on an individual's development, environmental enrichment (EE), on the other hand, can have therapeutic potential. In laboratory condition, EE aims to improve the welfare of the animal by including social or nonsocial features and EE stimulates HP neurogenesis [22]. The act of socializing itself can increase the level of monoamine NTs in mesolimbic structures of the brain. EE can protect animals from drug addiction by sensitizing limbic structures. In addition, rats given EE are less sensitive to the reinforcing effects of amphetamine [23]. Major developmental changes are triggered by some kind of stress that is associated with changes in the reward circuit’s neural pathways, including the HP. There have been reports of reductions in 5HT release, reduced 5HT tissue concentrations, and reduced synaptic activity in critical corticolimbic structures after exposure to stress in rodents [24, 25]. Social isolation is a good laboratory tool to induce stress, since in animals usually involve depriving them of social contact with their own species and studying how their brains and behaviors change during and after social isolation. The effects of social isolation on the brain and behavior are significant, especially during development. Moreover, animal models of social deprivation demonstrated that neurobiological mechanisms and development are deprived of stimuli that are crucial for their maintenance and development [26]. During critical developmental periods, genetic processes and environmental factors interact to stabilize certain specific traits of an organism [27]. During adolescence 5HT levels vary differently in different brain regions. There is an increase in serotonergic activity in the prefrontal cortex, but a decrease in serotonergic activity in the HP [26]. Rodent animal models reported, that is deprivation of social contact for approximately 1–3 weeks leads to anxiety-like behaviors and reduction in cell proliferation and neurogenesis are observed [26, 28].

Based on the above, the aim of this study was to investigate the impact of early postnatal MA exposure (PND 1–12) on changes in 5HT levels in the HP at 3 stages of adolescence (PND 28, 35, 45). Given the importance of the environment in which the young organism develops, we also examined the effect of hosing before weaning (EE vs standard) and after weaning (group housing vs social isolation).

## Methods

### Animals

The procedures used in this study were reviewed and approved by the Institutional Animal Care and Use Committee and meet the Czech Government Requirements put forth under the Policy of Humans Care of Laboratory Animals (No. 86/609/EEC) and with the subsequent regulations of the Ministry of Agriculture of the Czech Republic.

Adult female Wistar rats were purchased from Velaz (Prague, Czech Republic and bred by Charles River Laboratories International, Inc.). Rats were housed in a temperature-controlled (22–24 °C) room using a standard 12 h light/dark cycle and were left undisturbed for 1 week before fertility determination. Food and water were available ad libitum during that period. For determination of estrous cycle phase female rats were smeared using vaginal lavage. At the estrous phase females were housed overnight with sexually matured males. Determination of fertilization was performed by smearing of females for presence of sperm [13, 29]. The day after birth, the number of pups in each litter was adjusted to 12. Pups were randomly assigned to MA-treated (MA) groups and saline-treated (SA) control groups.

### Drugs

Physiological saline (0.9% NaCl) and d-Methamphetamine hydrochloride were purchased from Sigma-Aldrich (Czech Republic). MA was injected subcutaneously at a 5 mg/ml/kg dose to rat pups (direct) or rat lactating mothers (indirect) during PND 1–12, and control SA rats were given the same volume of SA. Administration of substances was held during morning hours. This dose is dose commonly used in our laboratory since 2005, because it induces the concentration in the blood comparable to human studies [30].

### Experimental design and procedure

**Fig. 1:**
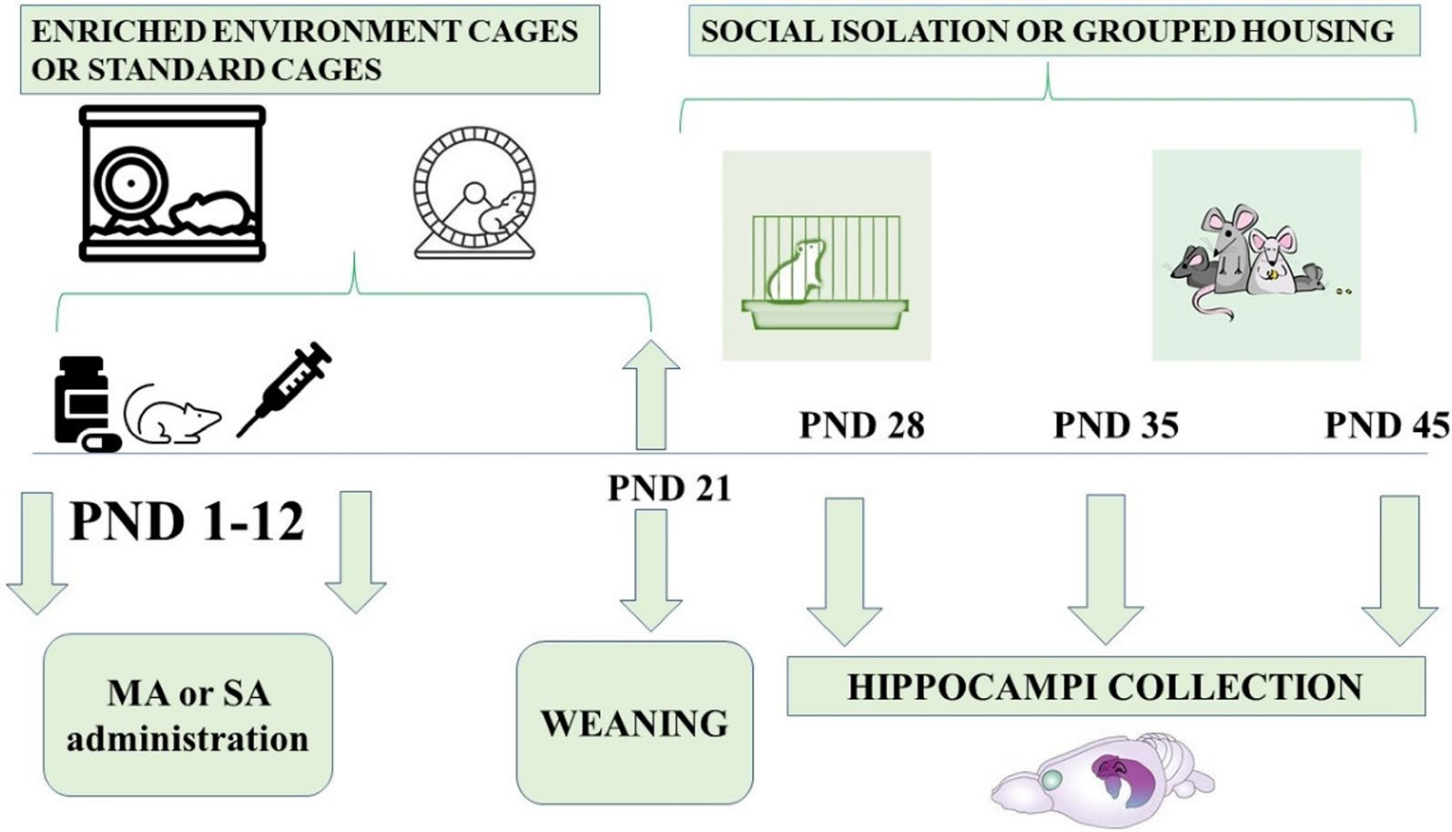
Timeline of the experiment. Timeline demonstrate the structure of the experiment

In this experiment, we studied the effect of two different methods of postnatal MA administration: direct—subcutaneous administration to pups on postnatal days (PND) 1–12, indirect—subcutaneous administration to mothers on PNDs 1–12, so that pups received the drug via breast milk. During the period before weaning (PND 1–21), pups were exposed to a standard environment (i.e., standard cages; L:39 × W:24 × H:18) used in our laboratory or to an enriched environment (EE) with larger cages (L:51 × W:42 × H:41) containing various toys. Pups were divided into groups according to the environment in which they were raised. On PND 21, the pups were weaned from mothers and divided into two different groups. Housed together—housed in groups of 4 (natural for rats as social animals) (L:51 × W:42 × H:41). Housed separately—rats were housed separately, one animal per cage (L:39 × W:24 × H:18) which is thought to be a stressor. The experimental timeline is shown in Fig. 1.

In total, 384 male rat pups were used in the study. As shown in Table 1, there were twelve groups based on the age when sacrificed (i.e., PND 28—early adolescence, PND 35—mid-adolescence, PND 45—late adolescence) and based on the treatment (MA vs. SA), drug application (direct vs. indirect), housing before weaning (standard cage vs. EE), and housing postweaning (group vs. separate). Eight rats were used in each group. The animals were left undisturbed until the day of sacrifice. In the present experiment, only males were tested because of the complications linked with the hormonal cycle in females. Differences which are possibly related to female hormones were previously reported by our team [31, 32].

**Table 1:** Overall, groups of animals were used in this experiment

PND of sacrifice	Form of exposure	Environment before weaning	Housing after weaning	Treatment
28, 35 or 45	Direct/indirect	Standard/EE	Group/separate	MA/SA

Sixty-four animals were used per one-period group. Animals were exposed to MA or SA as a control

### Immunoanalyses

On the respective days (PND 28, 35, and 45), the animals were peritoneally anesthetized with an overdose of chloralhydrate (400 mg/kg, Sigma-Aldrich) and given an intracardial perfusion of heparinized saline. The hippocampi were removed, snap frozen on dry ice, and stored at − 70 °C for further processing. During processing, the samples were homogenized in physiological saline containing 1 mM EDTA and 4 mM sodium metabisulfite (Sigma Aldrich) for a final concentration of 100 mg/ml. The homogenates were centrifuged at 10,000*g* in a cooled microcentrifuge for 10 min; the supernatants were aliquoted and stored frozen at − 70 °C until assayed. For 5HT quantitation ELISA kits were used (BAE-5900, LDN, Nordhorn Germany). Prior to assaying, we performed a serial dilution of selected samples using BAE-5941 as the diluent. Assays used samples diluted 20-times, and analyses were performed according to the manufacturer´s instructions. The absorbance was read at 450 nm on an 800™ TS microplate Absorbance Reader (BioTek). The intra-assay coefficient of variation (CV) was 8.96%, and the inter-assay CV was 3%. Protein concentrations were estimated using the Bradford method.

### Statistical analyses

A three-way ANOVA (treatment × preweaning environment × postweaning housing) was performed to measure the statistical significance, which was done separately for each of the different ages and forms of exposure. A one-way ANOVA was used to measure the statistical significance of the difference between controls, which we referred as standard group, on individual postnatal days. The Tukey post hoc test was used for multiple comparisons between groups. For statistical analyses Tibco Statistica software and Graph Pad Prism 8 were used. Differences were considered significant if *p* < 0.05. All values stated in the results are means ± SEM.

## Results

### Level of 5HT on PND 28

**Fig. 2:**
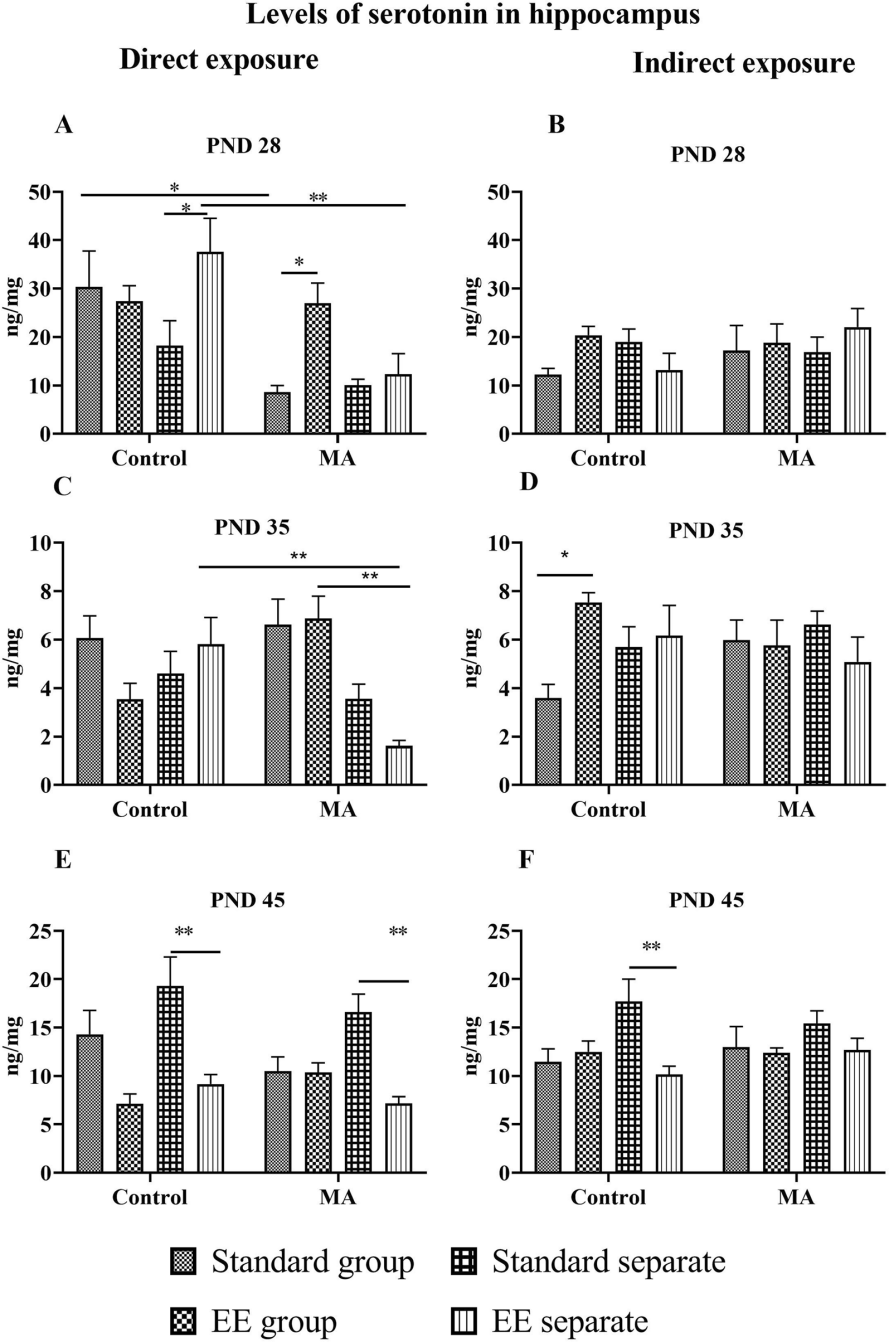
Levels of serotonin in hippocampus on all postnatal days. Graph A shows levels of 5HT on PND 28 after direct exposure, graph B shows levels of 5HT on PND 28 after indirect exposure. Graph C shows levels of 5HT on PND 35 after direct exposure, graph D shows level of 5HT on PND 35 after indirect exposure. Graph E shows levels of 5HT on PND 45 after direct exposure and graph F shows levels of 5HT on PND 45 after direct exposure. Levels of 5HT is expressed in ng of 5HT/mg of total protein in hippocampus. Values are means ± SEM (n = 8) *p < 0.05; **p < 0.01

Regarding 5HT on PND 28 after direct exposure, factors of significance were treatment [F_(1,56)_ = 22.48; *p* = 0.0001], environment [F_(1,56)_ = 9.638; *p* = 0.030], and interaction between all three factors [F_(1,56)_ = 14.34; *p* = 0.0004]. We observed significantly lower levels of 5HT in the MA standard group compared to the control standard group (*p* = 0.0097). Exposure to EE alone did not affect basal 5HT levels in SA treated group but enhanced the partially muted 5HT levels in SA treated separated animals (*p* = 0.0398). Low 5HT levels in MA-treated animals were also significantly boosted by the EE (*p* = 0.0263), but 5HT levels in the MA EE group separated rats remained low, without any EE effects. The difference between MA EE separate and control EE separate was also significant (0.0037). In terms of indirect exposure, no significant differences were observed (Fig. 2A, B).

### Levels of 5HT on PND 35

Regarding 5HT levels on PND 35 after direct exposure, the main significant effect was housing [F_(1,56)_ = 12.80; *p* = 0.0007]; there was also interaction between treatment and housing [F_(1,56)_ = 18,10; *p* = 0.0001] and an interaction between all three factors [F_(1,56)_ = 9.167; *p* = 0.0095]. We observed significantly higher levels of 5HT in the control EE separate compared to the MA EE separate (*p* = 0.0055) and in the MA EE group compared to the MA EE separate group (*p* = 0.0011). In terms of indirect exposure, the only effect of significance was an interaction between environment and treatment [F_(1,56)_ = 8.180; *p* = 0.0094]; we observed significantly higher levels of 5HT in the control EE group compared to the control standard group (*p* = 0.014) (Fig. 2C, D).

### Levels of 5HT on PND 45

Regarding 5HT levels on PND 45, after direct exposure, factors of significance were environment [F_(1,56)_ = 32.42; *p* = 0.0001], housing [F_(1,56)_ = 4.795; *p* = 0.0327] as well as interaction between environment and housing [F_(1,56)_ = 6.638; *p* = 0.0126]. We observed significantly higher levels of 5HT in the control standard separate compared to the control EE separate group (*p* = 0.0025) and in the MA standard separate compared to the MA EE separate (*p* = 0.0059). In terms of indirect exposure, the significant factors of significance were environment [F_(1,56)_ = 7.539; *p* = 0.0081] as well as an interaction between environment and housing [F_(1,56)_ = 9.056; *p* = 0.0039]. We observed a significantly higher level of 5HT in the control SA standard separate compared to the control EE (*p* = 0.0082) (Fig. 2E, F).

### Fluctuation of 5HT during development

#### Direct exposure

**Fig. 3:**
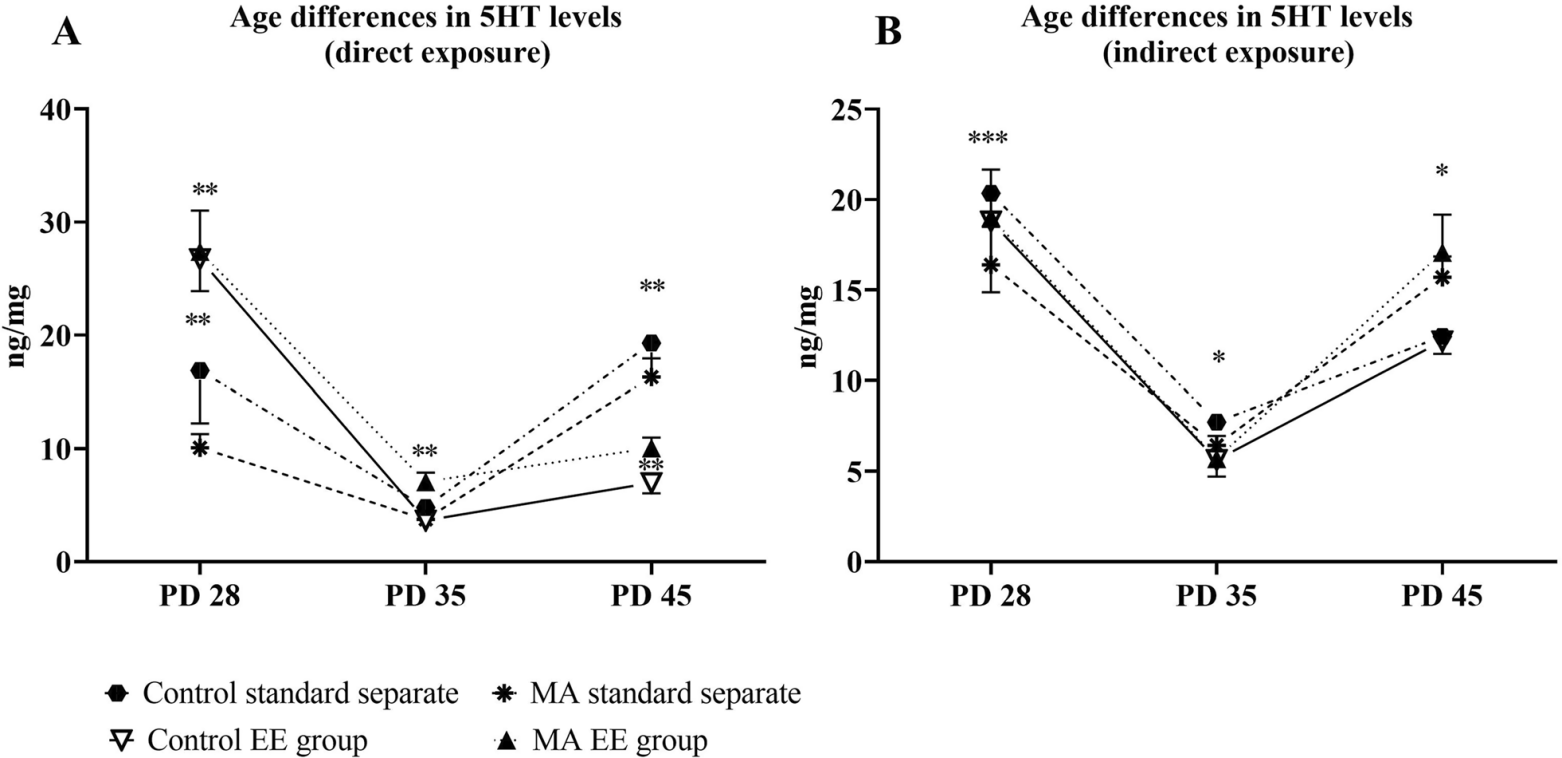
Fluctuation of 5HT during three different stages of adolescence. EE group represents best environmental conditions and standard separate worst environmental conditions. Graph A shows levels of 5HT after direct exposure and graph B shows levels of 5HT after indirect exposure. Levels of 5HT are expressed in ng of 5HT/mg of total protein in hippocampus. Values are means ± SEM *p < 0.05; **p < 0.01; ***p < 0.001

Within this form of exposure, we obtained many significant differences depending on period of adolescence. Two-way ANOVA of repeated measures showed age difference as significant factor [F_(2,56)_ = 49.99, *p* = 0.0001] as well as interaction [F_(6,56)_ = 37.46, *p* = 9.519]. Levels of 5HT within control EE group were significantly higher on PND 28 than on PND 35 (*p* = 0.0001) as well as PND 45 (*p* = 0.0012). Within control standard separate, these levels were significantly higher on PND 45 than PND 35 (*p* = 00034). In terms of MA EE group, levels were significantly higher on PND 28 in comparison with PND 35 (*p* = 0.0031) and PND 45 (0.0042). Levels within MA standard separate were significantly highest on PND 28 than on PND 35 (*p* = 0.0042), but significantly lower than PND 45 (*p* = 0.0184). These levels were also significantly higher on PND 45 in comparison with PND 35 (*p* = 0.0008). In analysis of differences between levels on PNDs in general, we obtained significant between all PNDs. Levels on PND 28 were significantly higher than on PND 35 (*p* = 0.0001) and PND 45 (*p* = 0.0318) and significantly higher on PND 45 than PND 35 (*p* = 0.0001). Fluctuation of 5HT after direct exposure is shown in Fig. 3A.

#### Indirect exposure

In terms of indirect exposure, Two-way ANOVA of repeated measures showed age difference as significant factor [F_(2,56)_ = 42.84, *p* = 0.0001]. Levels of 5HT were significantly higher in EE standard group on PND 28 than on PND 35 (*p* = 0.0006) and on PND 45 (*p* = 0.0205). Level on PND 45 were significantly higher in comparison with PND 35 (*p* = 0.0107). Control standard separate animals had significantly highest levels on PND 28 in comparison with PND 35 (*p* = 0.0028) and levels on PND 35 were significantly lower than levels on PND 45 as well (*p* = 0.0093). Within MA EE group, levels were significantly higher on PND 28 in comparison to PND 35 (*p* = 0.0288) and levels on PND 35 were significantly lower in comparison to PND 45 (*p* = 0.0001). In terms of MA standard separate, levels on PND 28 were significantly higher than on PND 35 (*p* = 0.0175) and levels on PND were significantly lower than on PND 45 (*p* = 0.0001). In analysis of differences between levels on PNDs in general, we obtained significant between all PNDs. Levels on PND 28 were significantly higher than on PND 35 (*p* = 0.0001) and PND 45 (*p* = 0.0408) and significantly higher on PND 45 than PND 35 (*p* = 0.0001). Fluctuation of 5HT after indirect exposure is shown in Fig. 3B.

### Comparison of differences in 5HT levels in control standard groups and MA standard groups based on postnatal day

**Fig. 4:**
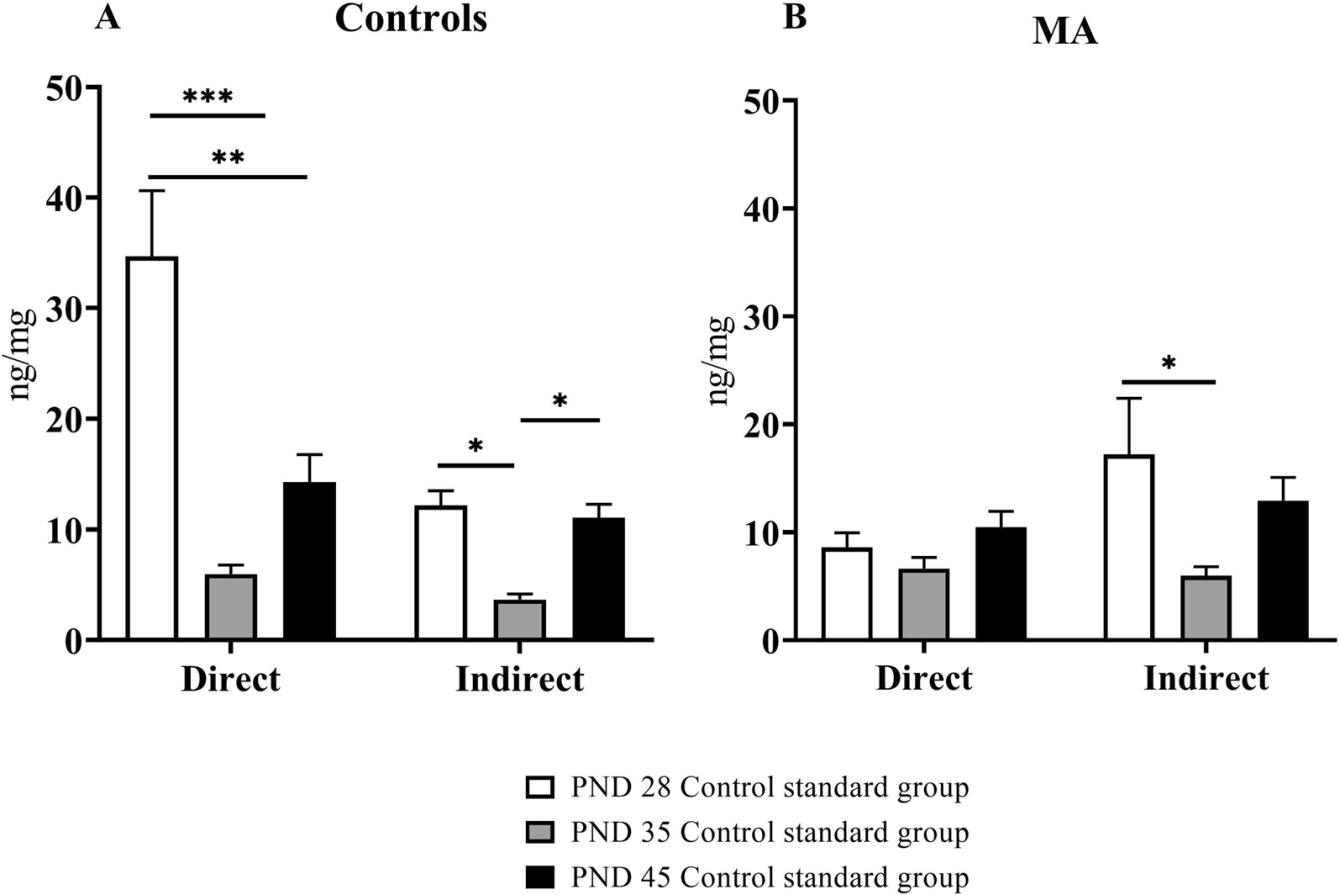
Comparison of levels of 5HT in standard/group between respective postnatal days. This Figure shows mostly the effect of MA, because group housing in standard cages without EE is standard housing condition in our laboratory. Graph A shows levels of 5HT after direct exposure and indirect exposure in standard control group. Graph B shows levels of 5HT after direct and indirect exposure in MA standard group. Levels of 5HT are expressed in ng of 5HT/mg of total protein in hippocampus. Values are means ± SEM *p < 0.05; **p < 0.01; ***p < 0.001

In Fig. 4A significant differences in the levels of 5HT after direct exposure are demonstrated at different ages of adolescence [F_(2,21)_ = 11.69; *p* = 0.0003]. Rats on PND 28 had higher 5HT levels than rats on PND 35 (*p* = 0.0015), and rats on PND 45 (*p* = 0.0259). Animals indirectly exposed of SA induces different effects on 5HT depending on stage of adolescence [F_(2,21)_ = 18.96; *p* = 0.0001]. Rats on PND 35 had lower 5HT levels than rats on PND 28 (*p* = 0.0002) and on PND 45 (*p* = 0.0002).

In Fig. 4B there were differences in the levels of 5HT according to age [F_(2,38)_ = 3.448; *p* = 0.0421]. Significant differences between levels appeared only among indirectly exposed animals between PND 28 and PND 35 exposed to MA (*p* = 0.0129).

## Discussion

In the current study, we explored the effects of direct and indirect exposure to MA on 5HT in the HP on PNDs 28, 35, and 45 in male rats relative to environmental conditions (EE vs. stress-inducing separate housing). To the best of our knowledge, this is the one of few studies of its kind, and therefore, it is difficult to compare our results with existing data.

### Effect of MA treatment

Most of the experiments related to MA exposure are focused on adult animals; the effect of MA on young brains is incomplete. Studies by Schaefer et al. [33, 34] reported that the effect of regular neonatal MA treatment on the activity of 5HT during the neonatal period can be detected very soon after treatment, and serotonergic neurotransmission declines during ontogenesis. The authors reported an immediate 5HT decline in the HP 1 day after a 5-day (PND 11–15) or 10-day (PND 11–20) MA administration dosing schedule of 10 mg/kg; 4×/day. On day 30, the effect was no longer visible, suggesting that the MA effect was only transient. In our experiment, when using a repeated daily dose of MA (5 mg/kg) on PNDs 1–12, we observed evident inhibition of 5HT that was still present on PND 28, i.e., 16 days after the discontinuation of the treatment, although on PND 35 and 45 the effect was no longer detectable. It seems that the timing and method of MA administration are crucial due to the different susceptibility of 5HT neurons during postnatal development. It must be emphasized that the effect was manifested only after direct administration.

Vorhees et al. [35, 36] described effect of MA-treated rats on PND 1–10 and 10–20 with dose of 30 mg/kg and according to these findings, MA exposure during both of these periods lead to impaired performance in a complex multiple-T water maze. Interestingly, only later period of MA exposure impaired learning in the Morris water maze. This study may not be related to 5HT changes, but it shows that the effects of MA are stage-dependent, and MA affects arousal and cognitive functions. It has been reported that MDMA (which also belongs to amphetamine group) self-administration leads to down-regulation of several 5HT receptor subtypes. Additionally, repeated exposure to MDMA by self-administration decreases tissue levels of 5HT and 5HT transporter densities [37]. As for the form of exposure, MA consumed via breast milk, even though consumed continuously throughout the day, was not enough to evoke an effect like daily injections.

Given the inhibiting effect of MA on 5HT production and neurogenesis is short lasting during the developmental stage, we can speculate that natural neuronal growth overcame the early effect of MA. Some data suggest more resistance to MA in developing brains compared to adult brains, indicating differences during development that need to be explored [38]. Data on the effect of MA on adolescent rat brains remain scarce, and there are no studies describing the immediate effects of postnatal MA administration. Additionally, research on MA administration with altered environment is also lacking.

### Effect of social environment

Dysregulation of NT interactions and other molecular and neurochemical impairments were found in brain regarding to social separation [39]. Interestingly, according to the study by Fosnocht et al. [40], social isolation during adolescence led to vulnerability to cocaine seeking behavior and alter behavioral responses to cocaine later in adulthood. It also altered the sensibility of reward circuit in the brain. Conversely, EE has been shown to produce an opposite effect. It was reported that EE can stimulate various biochemical, and functional changes in the HP, especially the network connectivity, as well as the developing of new neurons in the dentate gyrus of mice exposed to an EE compared with standard housing environment [41]. EE was shown to have therapeutic potential when introducing after exposure to drugs and the development of drug-taking habits in adults [42]. A study by Sbrini et al. [43] reported that during 1 month of EE exposure the anhedonia and anxiety-like phenotype characteristics were normalized in animal model of depression. Their data confirmed the ability of EEs to restore behavioral and molecular changes, thus supporting that EE may likely be one of the non-pharmacological approaches to treating mood disorders [43].

A huge impact of EE has been reported on HP neurogenesis. According to reports, EE affects gene expression in cells, alters protein production, and models hormone, neurotransmitter, and neurotrophic factor biochemical processes [22, 44]. Studies report that rodents living in EE show greater cerebral weight and length, increased cortical depth and changes in CA1 region of HP as well as increases in dendritic spines and branching, synaptic connections, and neural cell size [22]. However, the effect of EE on 5HT levels has not been well documented, especially in connection with MA and the adolescent brain. Results of our study show the effect of EE on 5HT levels, especially in combination with housing conditions and MA. In SA-treated PND 28 pups, the clearly improved 5HT levels in pups subjected to separation stress suggesting that enhanced external stimuli can compensate for the effects of isolation stress. A similar response was found in MA-treated pups, where suppressed 5HT levels were restored by the EE. However, the combination of MA and separate housing maintained reduced 5HT levels, and the EE remained without and beneficial effect. Given that EE improved 5HT levels in control separated pups but not in MA-treated pups, it appears that the combination of these two negative factors, i.e. separation and MA, had such robust effects that could not be overcame by EE. Data from other PNDs showed different situation, therefore it is possible different exposures, relative to PNDs, may have affected neurogenesis and associated levels of 5HT. As mentioned above, the levels of 5HT were very low in all groups compared to those at PND 28. Moreover, we found that 5HT levels were paradoxically lower in control animals exposed to EEs than in standard environments. Even though it was an unexpected finding, it can be ascribed to some discomfort associated with removal from the EE environment. On the contrary to PND 28 on other PNDs MA itself did not affect 5HT levels and the exposure to EE remained without any further effect. However, EE potentiated the inhibitory effect caused by isolation stress. On PND 45, rats after early exposure to EE showed lower levels of 5HT than those housed under standard conditions in the SA-treated non-stressed rats and rats with separation stress. These findings underline the possibility of discomfort (withdrawal effect) in rats caused by the lack of EE after weaning. Another researchers [45] studied the effect of stress in adult animals on HP-dependent learning, memory performance and several NT levels [45]. Stress was demonstrated by barren cages housing in comparison to standard cages. Their research reported that 5HT levels in HP were decreased by MA in comparison to SA, but stressor environment did not seem to have eminent effect on these levels in comparison to standard environment. Although it is very important to highlight the fact, that all mentioned studied were performed mostly on adult animals with different doses of MA, type of injection and different type and duration of stress exposure.

### Effect of PND in respect of drugs and environment

Our results show highest 5HT levels on PND 28 and lowest on PND 35. Control pups injected with SA (direct exposure) showed higher levels of 5HT in each time intervals compared with non-treated, intact pups (when only mothers were exposed—indirect exposure). These differences are in line with our earlier findings showing that SA injection alone could have various effects and these results also propose that behavior of rats can be affected not only by drugs but the injection itself may have effects associated with repeated stress [30]. As previously described, the developmental fluctuating levels of 5HT during ontogenesis results from physiological reorganization of the developing serotonergic innervation [6, 46]. Nevertheless, we monitored only 5HT levels and did not investigate these changes in more details, our result of these levels correlates with previously described changes in 5HT development. Low 5HT activity in adolescence may lead to common adolescent behaviors such as hypersensitivity, impulsivity, increased anxiety, as well as onset of various drug abuse as coping mechanism. This indicates that 5HT neurotransmission is subjected to eminent remodeling from early postnatal development through adolescence into adulthood and is sensitive to disruption by drugs and other stressors as well [8].

### Effect of injection (direct vs indirect exposure)

We did not observe many significant differences in indirect MA exposure. Our previous experiments demonstrated differences between direct versuss indirect MA administration on rat performance on various behavioral tests, which supports our present results [47, 48]. We hypothesize that direct early postnatal MA injection exhibits an instantaneous effect on the body while MA injected through breast milk is slowly absorbed into the body of pups [13]. During indirect MA exposure, MA is partly metabolized in the mother’s body. The half-life of MA in rats is reported to be around 70 min [49, 50]. Moreover, MA-treated mothers display more self-care activities and consequently pay less attention to the litter immediately following drugs exposure [51]; thus, pups may not have a chance to suck until the effect of the drug on the mother has diminished, which exposes the pups to lower MA doses than after direct MA injections. A study showed that maternal care differences during the first week of postnatal life could influence HP development and function [52].

Serotonin is often associated with anxiety, but its role in this disorder is not yet fully understood [53]. Some studies showed that increased serotonergic activity leads to enhanced anxiety behavior [54], while others found the opposite [55, 56]. These paradoxical results suggest different roles of various 5HT receptor in development of anxiety and depression [54]. Anxious behavior is also related to stress exposure. In the present study, stress is represented by social separation as well as exposure to MA in the early postnatal period. Although behavioral experiments are not included in this paper, we can compare our results with our previous publications.

Previous behavioral studies from our laboratory showed that prenatal exposure to MA and stress, have no effect on anxiety behavior in adult male rats as measured by open-field and elevated plus maze tests, as well as did not prove any anxiogenic effects in postnatally stressed rats in adulthood [57]. On the other hand, postnatal exposure to MA via breast milk in the pre-weaning period (PND 1–21) decreases locomotion and exploratory behavior in and increases anxiety behavior as shown in the study Hruba et al. [31]. Because there are data demonstrating that MA is neurotoxic to the dopaminergic [58] and serotoninergic systems [59, 60], it is possible that depletion of DA and 5HT causes a decrease in locomotion activity and an increase in anxiety behavior in rats postnatally exposed to MA. These results may also be explained by changes in maternal behavior, such as impaired maternal care, reduced active nursing, or care of pups [31]. Another study from our laboratory showed a significant reduction in the reactivity of the adrenal cortex of adult male rats to stress following neonatal exposure to social and/or physical stressors [61]. In addition, animals exposed to stress in early life (separation from the mother or cold water stress) show reduced parameters of anxiety-like behavior in adulthood [57]. On the other hand, a significant reduction in plasmatic oxytocin within postnatally stressed groups in control animals as well as prenatally exposed to MA [62]. Neonatal exposure to social and physical stress accelerated the habituation process to familiar environments and reduced cognitive processes in relation to increased novelty anxiety in adult male rats in the Holubova et al. (2018) study [63].

Our results show that both MA and environment (including stress) affect 5HT levels, depending on both the age of the offspring and the method of application (direct or indirect). In the future, we plan to investigate the degree of influence of the different factors behind the different results of direct and indirect drug application (injection vs. breast milk) that may result in different concentration of the drug in rat pup circulation.

## Conclusions

This study provides results that shed light on the effects of postnatal MA exposure and that environmental and social factors can influence its effects. In summary, our results show that daily administration of low dose of MA in the early postnatal period (PND 1 to PND 12) has a prolonged effect that leads to inhibition of 5HT production in the HP for up to 16 days after termination of injections (PND 28). Our results also showed that MA effects disappeared in the later stages of adolescence. This finding extends our knowledge of how MA affects the developing brain and demonstrates the brain’s enhanced sensitivity to MA exposure during the early postnatal period. We found that 5HT levels that had been muted in response to separation stress or MA were restored in rats that had also been exposed to an EE from birth until weaning (PND 28). This showed the beneficial effects of an EE. The EE could not, however, overcome the combined effects of both treatments (i.e., separation stress + MA). In older adolescent animals, the EE exerted an opposite effect, i.e., it lowered 5HT levels after separation stress or MA exposure, most likely because of a long-lasting withdrawal effect associated with ending exposure to the EE after weaning.

The longevity of the effect of EE is therefore still not fully understood, especially in relation to other negative factors affecting postnatal development, such as drug exposure. Our future studies will focus on the long-term effects of the positive impact of EE in adult animals pre- or early postnatally exposed to MA.

## Data Availability

The authors declare that the data supporting the findings of this study are available from the corresponding author upon reasonable request.

## Abbreviations

EE: Enriched environment
CNS: Central nervous system
HP: Hippocampus
MA: Methamphetamine
NT: Neurotransmitter
PND: Postnatal day
SA: Saline
5HT: Serotonin
Sc: Subcutaneously

## References

[1] Cechova B, Slamberova R (2021). Methamphetamine, neurotransmitters and neurodevelopment. Physiol Res.

[2] Jayanthi S, Daiwile AP, Cadet JL (2021). Neurotoxicity of methamphetamine: main effects and mechanisms. Exp Neurol.

[3] Bellia F, Suarez A, D'Addario C, Pautassi RM, Fabio MC (2021). Transient serotonin depletion at adolescence, but not at early infancy, reduced subsequent anxiety-like behavior and alcohol intake in female mice. Psychopharmacology.

[4] Park HS, Kim TW, Park SS, Lee SJ (2020). Swimming exercise ameliorates mood disorder and memory impairment by enhancing neurogenesis, serotonin expression, and inhibiting apoptosis in social isolation rats during adolescence. J Exerc Rehabil.

[5] Ziemens D, Touma C, Rappeneau V (2022). Neurobiological mechanisms modulating emotionality, cognition and reward-related behaviour in high-fat diet-fed rodents. Int J Mol Sci.

[6] Crews F, He J, Hodge C (2007). Adolescent cortical development: a critical period of vulnerability for addiction. Pharmacol Biochem Behav.

[7] Anton-Toro LF, Bruna R, Del Cerro-Leon A, Shpakivska D, Mateos-Gordo P, Porras-Truque C (2022). Electrophysiological resting-state hyperconnectivity and poorer behavioural regulation as predisposing profiles of adolescent binge drinking. Addict Biol.

[8] Semple BD, Blomgren K, Gimlin K, Ferriero DM, Noble-Haeusslein LJ (2013). Brain development in rodents and humans: identifying benchmarks of maturation and vulnerability to injury across species. Prog Neurobiol.

[9] Jacob FD, Habas PA, Kim K, Corbett-Detig J, Xu D, Studholme C (2011). Fetal hippocampal development: analysis by magnetic resonance imaging volumetry. Pediatr Res.

[10] Kim EJ, Pellman B, Kim JJ (2015). Stress effects on the hippocampus: a critical review. Learn Mem.

[11] Trakhtenberg EF, Goldberg JL (2012). The role of serotonin in axon and dendrite growth. Int Rev Neurobiol.

[12] Rice D, Barone S (2000). Critical periods of vulnerability for the developing nervous system: evidence from humans and animal models. Environ Health Perspect.

[13] Petrikova-Hrebickova I, Sevcikova M, Slamberova R (2021). The impact of neonatal methamphetamine on spatial learning and memory in adult female rats. Front Behav Neurosci.

[14] Slamberova R (2019). Review of long-term consequences of maternal methamphetamine exposure. Physiol Res.

[15] Zhang Y, Gong F, Liu P, He Y, Wang H (2021). Effects of prenatal methamphetamine exposure on birth outcomes, brain structure, and neurodevelopmental outcomes. Dev Neurosci.

[16] Slamberova R, Pometlova M, Schutova B, Hruba L, Macuchova E, Nova E (2012). Do prenatally methamphetamine-exposed adult male rats display general predisposition to drug abuse in the conditioned place preference test?. Physiol Res.

[17] Yamamotova A, Slamberova R (2012). Behavioral and antinociceptive effects of different psychostimulant drugs in prenatally methamphetamine-exposed rats. Physiol Res.

[18] Bernaskova K, Matejovska I, Slamberova R (2011). Postnatal challenge dose of methamphetamine amplifies anticonvulsant effects of prenatal methamphetamine exposure on epileptiform activity induced by electrical stimulation in adult male rats. Exp Neurol.

[19] Slamberova R, Pometlova M, Macuchova E, Nohejlova K, Stuchlik A, Vales K (2015). Do the effects of prenatal exposure and acute treatment of methamphetamine on anxiety vary depending on the animal model used?. Behav Brain Res.

[20] Rokyta R, Yamamotova A, Slamberova R, Franek M, Vaculin S, Hruba L (2008). Prenatal and perinatal factors influencing nociception, addiction and behavior during ontogenetic development. Physiol Res.

[21] Spear LP (2007). Assessment of adolescent neurotoxicity: rationale and methodological considerations. Neurotoxicol Teratol.

[22] Bayne K (2018). Environmental enrichment and mouse models: current perspectives. Anim Model Exp Med.

[23] Zentall TR (2021). Effect of environmental enrichment on the brain and on learning and cognition by animals. Animals (Basel).

[24] Mahar I, Bambico FR, Mechawar N, Nobrega JN (2014). Stress, serotonin, and hippocampal neurogenesis in relation to depression and antidepressant effects. Neurosci Biobehav Rev.

[25] Yao X, Yang C, Wang C, Li H, Zhao J, Kang X (2022). High-fat diet consumption in adolescence induces emotional behavior alterations and hippocampal neurogenesis deficits accompanied by excessive microglial activation. Int J Mol Sci.

[26] Orben A, Tomova L, Blakemore SJ (2020). The effects of social deprivation on adolescent development and mental health. Lancet Child Adolesc Health.

[27] Nelson CA, Gabard-Durnam LJ (2020). Early adversity and critical periods: neurodevelopmental consequences of violating the expectable environment. Trends Neurosci.

[28] Luikinga SJ, Kim JH, Perry CJ (2018). Developmental perspectives on methamphetamine abuse: exploring adolescent vulnerabilities on brain and behavior. Prog Neuropsychopharmacol Biol Psychiatry.

[29] Sevcikova M, Petrikova I, Slamberova R (2020). Methamphetamine exposure during the first, but not the second half of prenatal development, affects social play behavior. Physiol Res.

[30] Slamberova R, Nohejlova K, Ochozkova A, Mihalcikova L (2018). What is the role of subcutaneous single injections on the behavior of adult male rats exposed to drugs?. Physiol Res.

[31] Hruba L, Schutova B, Slamberova R (2012). Sex differences in anxiety-like behavior and locomotor activity following prenatal and postnatal methamphetamine exposure in adult rats. Physiol Behav.

[32] Slamberova R, Mikulecka A, Pometlova M, Schutova B, Hruba L, Deykun K (2011). Sex differences in social interaction of methamphetamine-treated rats. Behav Pharmacol.

[33] Schaefer TL, Skelton MR, Herring NR, Gudelsky GA, Vorhees CV, Williams MT (2008). Short- and long-term effects of (+)-methamphetamine and (+/-)-3,4-methylenedioxymethamphetamine on monoamine and corticosterone levels in the neonatal rat following multiple days of treatment. J Neurochem.

[34] Schaefer TL, Grace CE, Gudelsky GA, Vorhees CV, Williams MT (2010). Effects on plasma corticosterone levels and brain serotonin from interference with methamphetamine-induced corticosterone release in neonatal rats. Stress.

[35] Vorhees CV, Ahrens KG, Acuff-Smith KD, Schilling MA, Fisher JE. Methamphetamine exposure during early postnatal development in rats: II. Hypoactivity and altered responses to pharmacological challenge. Psychopharmacology (Berl). 1994;114(3):402–8. 10.1007/BF02249329 7855198

[36] Vorhees CV, Ahrens KG, Acuff-Smith KD, Schilling MA, Fisher JE. Methamphetamine exposure during early postnatal development in rats: I. Acoustic startle augmentation and spatial learning deficits. Psychopharmacology (Berl). 1994;114(3):392–401. 10.1007/BF02249328 7855197

[37] Schenk S, Highgate Q (2021). Methylenedioxymethamphetamine (MDMA): serotonergic and dopaminergic mechanisms related to its use and misuse. J Neurochem.

[38] Buck JM, Morris AS, Weber SJ, Raber J, Siegel JA (2017). Effects of adolescent methamphetamine and nicotine exposure on behavioral performance and MAP-2 immunoreactivity in the nucleus accumbens of adolescent mice. Behav Brain Res.

[39] Biggio F, Mostallino MC, Talani G, Locci V, Mostallino R, Calandra G (2019). Social enrichment reverses the isolation-induced deficits of neuronal plasticity in the hippocampus of male rats. Neuropharmacology.

[40] Fosnocht AQ, Lucerne KE, Ellis AS, Olimpo NA, Briand LA (2019). Adolescent social isolation increases cocaine seeking in male and female mice. Behav Brain Res.

[41] Ohline SM, Abraham WC (2019). Environmental enrichment effects on synaptic and cellular physiology of hippocampal neurons. Neuropharmacology.

[42] Galaj E, Barrera ED, Ranaldi R (2020). Therapeutic efficacy of environmental enrichment for substance use disorders. Pharmacol Biochem Behav.

[43] Sbrini G, Brivio P, Bosch K, Homberg JR, Calabrese F (2020). Enrichment environment positively influences depression- and anxiety-like behavior in serotonin transporter knockout rats through the modulation of neuroplasticity, spine, and GABAergic markers. Genes (Basel).

[44] Kempermann G (2019). Environmental enrichment, new neurons and the neurobiology of individuality. Nat Rev Neurosci.

[45] Gutierrez A, Jablonski SA, Amos-Kroohs RM, Barnes AC, Williams MT, Vorhees CV (2017). Effects of housing on methamphetamine-induced neurotoxicity and spatial learning and memory. ACS Chem Neurosci.

[46] Arrant AE, Jemal H, Kuhn CM (2013). Adolescent male rats are less sensitive than adults to the anxiogenic and serotonin-releasing effects of fenfluramine. Neuropharmacology.

[47] Hrebickova I, Sevcikova M, Macuchova E, Slamberova R (2017). How methamphetamine exposure during different neurodevelopmental stages affects social behavior of adult rats?. Physiol Behav.

[48] Hrebickova I, Sevcikova M, Nohejlova K, Slamberova R (2016). Does effect from developmental methamphetamine exposure on spatial learning and memory depend on stage of neuroontogeny?. Physiol Res.

[49] Melega WP, Cho AK, Harvey D, Lacan G (2007). Methamphetamine blood concentrations in human abusers: application to pharmacokinetic modeling. Synapse.

[50] Cho AK, Melega WP, Kuczenski R, Segal DS (2001). Relevance of pharmacokinetic parameters in animal models of methamphetamine abuse. Synapse.

[51] Sevcikova M, Hrebickova I, Macuchova E, Slamberova R (2017). The influence of methamphetamine on maternal behavior and development of the pups during the neonatal period. Int J Dev Neurosci.

[52] Liu D, Diorio J, Day JC, Francis DD, Meaney MJ (2000). Maternal care, hippocampal synaptogenesis and cognitive development in rats. Nat Neurosci.

[53] Sbrini G, Hanswijk SI, Brivio P, Middelman A, Bader M, Fumagalli F (2022). Peripheral serotonin deficiency affects anxiety-like behavior and the molecular response to an acute challenge in rats. Int J Mol Sci.

[54] Albert PR, Vahid-Ansari F, Luckhart C (2014). Serotonin-prefrontal cortical circuitry in anxiety and depression phenotypes: pivotal role of pre- and post-synaptic 5-HT1A receptor expression. Front Behav Neurosci.

[55] Blazevic S, Colic L, Culig L, Hranilovic D (2012). Anxiety-like behavior and cognitive flexibility in adult rats perinatally exposed to increased serotonin concentrations. Behav Brain Res.

[56] Mosienko V, Bert B, Beis D, Matthes S, Fink H, Bader M (2012). Exaggerated aggression and decreased anxiety in mice deficient in brain serotonin. Transl Psychiatry.

[57] Holubova-Kroupova A, Slamberova R (2021). Perinatal stress and methamphetamine exposure decreases anxiety-like behavior in adult male rats. Front Behav Neurosci.

[58] Seiden LS, Commins DL, Vosmer G, Axt K, Marek G (1988). Neurotoxicity in dopamine and 5-hydroxytryptamine terminal fields: a regional analysis in nigrostriatal and mesolimbic projections. Ann N Y Acad Sci.

[59] Buening MK, Gibb JW (1974). Influence of methamphetamine and neuroleptic drugs on tyrosine hydroxylase activity. Eur J Pharmacol.

[60] Hotchkiss AJ, Gibb JW (1980). Long-term effects of multiple doses of methamphetamine on tryptophan hydroxylase and tyrosine hydroxylase activity in rat brain. J Pharmacol Exp Ther.

[61] Holubova A, Stofkova A, Jurcovicova J, Slamberova R (2016). The effect of neonatal maternal stress on plasma levels of adrenocorticotropic hormone, corticosterone, leptin, and ghrelin in adult male rats exposed to acute heterotypic stressor. Physiol Res.

[62] Holubova A, Ponist S, Jurcovicova J, Slamberova R (2019). Different oxytocin responses to acute methamphetamine treatment in juvenile female rats perinatally exposed to stress and/or methamphetamine administration. Front Physiol.

[63] Holubova A, Lukaskova I, Tomasova N, Suhajdova M, Slamberova R (2018). Early postnatal stress impairs cognitive functions of male rats persisting until adulthood. Front Behav Neurosci.

